# Moisture Stability of Perovskite Solar Cells Processed in Supercritical Carbon Dioxide

**DOI:** 10.3390/molecules26247570

**Published:** 2021-12-14

**Authors:** Gilbert Annohene, Gary Tepper

**Affiliations:** Department of Mechanical and Nuclear Engineering, Virginia Commonwealth University, Richmond, VA 23284, USA; annoheneg@vcu.edu

**Keywords:** perovskite, photovoltaics, supercritical carbon dioxide

## Abstract

Performance degradation under environmental conditions currently limits the practical utility of perovskite-based solar cells. The moisture stability of CH_3_NH_3_PbI_3_ perovskite films and solar cells was measured during exposure to three different levels of relative humidity. The films were crystallized at two different temperatures with and without simultaneous exposure to supercritical carbon dioxide. The film crystallinity, optical absorption, and device photoconversion efficiency was measured over time for three relative humidity levels and both crystallization methods. It was determined that film crystallization in supercritical CO_2_ resulted in significant improvement in moisture stability for films processed at 50 °C, but negligible improvement in stability for films processed at 100 °C.

## 1. Introduction

Perovskite-based solar cells have emerged as a potentially disruptive photovoltaic technology. Perovskite refers to the ABX_3_ crystal structure (usually A is organic ammonium such as methylammonium, B is a cation such as lead, and X is a halide such as chlorine, or mixed halides). Perovskites have been explored for application in light emitting diodes (LEDs) [1,2,3], low-power transistors [4,5,6], and highly efficient photodetectors [7,8,9]. Halide perovskite materials (inorganic, organic-inorganic) are of interest in the solar cell field due to their long charge carrier diffusion length, high light absorption coefficient, tunable bandgap, large extinction coefficient, relatively low material cost, solution-based processing, and excellent photoelectric conversion efficiency [10,11,12,13,14,15,16,17,18,19]. These advantages make halide perovskites an ideal material in the field of photovoltaics and other optoelectronic devices. The power conversion efficiency (PCE) of perovskite is comparable with crystalline silicon solar cells and has increased from 3.8% in 2009 [20] to a certified record of 25.5% in 2019 [21].

The crystallinity of the perovskite thin film is paramount to achieve optimum device performance. High-quality crystalline perovskite films with large grains produce superior optoelectronic performance because of a lower recombination rate and longer carrier diffusion length. Post-deposition annealing is used to enable short-range interdiffusion of the perovskite precursor compounds on the substrate to facilitate conversion into photoactive perovskite crystals [22,23,24,25]. However, thermal annealing can produce impurities and intermediate phases at high temperatures, and is problematic when scaling to large device areas since temperature variations produce inhomogeneities in film crystallinity [26,27,28,29].

We recently introduced the use of supercritical fluids (SCF) for post-deposition annealing of perovskites [30,31,32]. Supercritical carbon dioxide was shown to be an anti-solvent to CH_3_NH_3_PbI_3_ perovskite and facilitated low-temperature crystallization by reducing the energy barrier for molecular diffusion. Supercritical carbon dioxide (scCO_2_) has a low critical point (31.2 °C, 7.38 Mpa or 1070.4 psi), no surface tension, liquid-like density, gas-like viscosity, and diffusivity and negligible solubility to the perovskite films. Trace amounts (less than 2%) of organic co-solvents could be added to the scCO_2_ to modify the solubility and produce various film morphologies and crystal orientations [31]. We have also reported the photovoltaic performance of CH_3_NH_3_PbI_3_ perovskite solar cells where the photoactive layer was annealed in scCO_2_ at 50 °C and achieved a PCE of 17.22% [32].

Although the photovoltaic performance of PSCs is outstanding, its major challenge to commercialization remains the stability of the crystals [33,34] under environmental conditions. Poor stability of PSCs is primarily due to degradation of the perovskite crystals and can be caused by many factors such as exposure to atmospheric oxygen and humidity [35,36,37,38], high temperature [39], illumination [40,41], and ion migration [42,43]. However, degradation by humidity remains the dominant factor reducing the stability of the perovskite materials. Several approaches have been adopted to stabilize halide perovskites, including compositional tuning [44,45,46], low dimensional perovskites [47,48], additives [49,50], use of a surface blocking layer [41,51,52], and enhancing the grain size.

For hybrid organic-inorganic perovskites, instability is due to the organic component, where the CH_3_NH_3_PbI_3_ decomposes into a lead iodide (PbI_2_) and methylammonium iodide (CH_3_NH_3_I). CH_3_NH_3_I gives up a proton to water, forming H_3_O^+^ by breaking the bond between the A and B molecules of the ABX_3_ perovskite structure [53,54,55]. Frost et al. explained a possible water-facilitated decomposition path shown in Equations (1)–(4) [56]. Exposure of the perovskite to moisture results in the organic iodide forming HI acid, which dissolves in water. Trace amounts of water are adequate to deprotonate the organic component to cause degradation. In addition, the exposure of the film to trace amounts of H_2_O results in partial decomposition until the byproducts reach equilibrium.

(1) $${\mathrm{CH}}_{3}{\mathrm{NH}}_{3}\mathrm{Pb}I_{3}(s)\;↔\;{\mathrm{CH}}_{3}{\mathrm{NH}}_{3}I\left(\mathrm{aq})+\mathrm{Pb}I_{2}(s\right)$$

(2) $${\mathrm{CH}}_{3}{\mathrm{NH}}_{3}I\left(\mathrm{aq})\;↔\;{\mathrm{CH}}_{3}{\mathrm{NH}}_{2}(\mathrm{aq})+\mathrm{HI}(\mathrm{aq}\right)$$

(3) $$4\mathrm{HI}(\mathrm{aq})+O_{2}\;↔\;2I_{2}(s)+2H_{2}O$$

(4) $$2\mathrm{HI}(\mathrm{aq})\;↔\;H_{2}\;+\;I_{2}(s)$$

In our initial studies, we demonstrated that annealing CH_3_NH_3_PbI_3_ perovskite thin films at low temperature in scCO_2_ resulted in high-quality films and devices with corresponding high photovoltaic efficiencies. In this paper, we investigate the humidity degradation rate of films and photovoltaic devices processed in scCO_2_. The effect of three levels of relative humidity (RH) (No exposure (<5% RH), 40% RH, and 60% RH) on perovskite film optical absorption, crystallinity and resulting device photo conversion efficiency (PCE) was measured for films processed in scCO_2_ in comparison to films annealed without scCO_2_.

## 2. Methods

### 2.1. Device Fabrication

All film and device fabrication procedures were reported previously [32]. Fluorine-doped tin oxide (FTO) glass substrates (Ossila, TEC 15, Sheffield, UK) of size 25 mm by 25 mm were used. The substrate was cleaned as follows; ultrasonic bath in 2% Hellmanex solution, rinse with deionized water, ultrasonic bath in isopropanol for 15 min, ultrasonic bath in acetone for 15 min, rinse acetone and isopropanol, dry the isopropanol with dry air and plasma cleaned for 15 min.

The titanium dioxide (TiO_2_) blocking layer (bl-TiO_2_) was formed by spin coating 0.15 M titanium diisopropoxide dis(acetylacetonate) (Sigma-Aldrich, 75 wt% in isopropanol) in 1-butanol (anhydrous, Sigma-Aldrich, 99.8%) onto the FTO glass substrate at 700 rpm for 8 s, 1000 rpm for 10 s and 2000 rpm for 40 s, followed by drying at 125 °C for 5 min [32,57]. The mesoporous TiO_2 (mp-TiO_2_) layer was deposited on the bl-TiO_2_ by spin-coating a TiO_2_ colloidal solution containing 0.6 g of TiO_2_ paste (30NR-D, Greatcell Solar) diluted in 5 mL of anhydrous ethanol solution at 2000 rpm for 20 s, followed by annealing at 540 °C for 1 h. The substrate was further treated with 20 mM aqueous TiCl_4_ (>98%, Sigma-Aldrich, Saint Louis, MO, USA) solution at 90 °C for 10 min, cleaned with deionized water and then sintered at 500 °C for 30 min [32,57].

CH_3_NH_3_PbI_3_ was synthesized by mixing a 1:1:1 molar ratio of 2.385 g of methyl ammonium iodide (CH_3_NH_3_I) (98%, Sigma Aldrich), 6.915 g of lead (II) iodide (PbI_2_) (99.9985%, Alfa Aesar, Ward Hill, MA, USA), and 1.063 mL of dimethyl sulfoxide (DMSO) (≥99.9%, anhydrous, Sigma Aldrich) in 9.484 mL of N,N-dimethylformamide (DMF) (≥99.8%, anhydrous, Sigma Aldrich) and 0.3 mL of diethyl ether (≥99.8% anhydrous, Sigma Aldrich). The solution was stirred for 1 h at room temperature and filtered with 0.2 μm syringe filter (Corning Inc., Corning, NY, USA). The solution was processed in an argon-filled glove box. The precursor solution was spin coated onto the substrate at 6000 rpm for 25 s. and 0.5 mL of diethyl ether was dripped onto the rotating surface 6 s into the spinning. For thermal annealed films, the substrate was placed on a hotplate at either 50 or 100 °C for 30 min. For scCO_2_ annealed films, the thin film was placed in a pressure vessel (Parr Instrument Pressure Reactor 4768) and a syringe pump (Teledyne ISCO Pump 260D) was used to pressurize the carbon dioxide at 1300 psi and 50 °C for 30 min. The substrates were then removed and blown with argon and dried in the glovebox [30,31,32].

A total of 65 µL of spiro-MeOTAD solution, which contained 72.3 mg spiro-MeOTAD (Sublimed, Ossila), 28.8 µL of 4-tert-butyl pyridine (Ossila), and 17.5 µL of lithium bis(trifluoromethanesulfonyl)imide (Li-TFSI) solution (520 mg Li-TSFI (Ossila) in 1 mL acetonitrile (Sigma–Aldrich, 99.8%) in 1 ml of chlorobenzene was spin-coated on the perovskite layer at 3000 rpm for 30 s [58,59]. Finally, the Silver (Ag) electrode was deposited using electron beam evaporation at a constant evaporation rate of 0.03 nm/s through a shadow mask resulting in an electrode thickness of approximately 100 nm. Home-made humidity chambers were used to expose the samples to the higher relative humidity levels (40 and 60%). For lower relative humidity (<5% RH), samples were placed in a dry desiccator kept in an argon filled glovebox.

### 2.2. Device Characterization

The crystallographic properties of the perovskite films were characterized by X-ray diffraction (XRD) measurements (PANalytical MPD X’pert Pro) using a Cu Kα (λ = 1.54 nm) radiation source that operated at 45 kV and 40 mA. The X-ray diffractograms were obtained at a scan rate of 0.01° s^−1^ for 2θ values between 10° and 60°. The surface coverage and grain size were obtained using a scanning electron microscope (SEM) (Hitachi SU-70 FE-SEM) at 20 kV, and analyzed using an ImageJ software package. Optical spectrometry (transmission, reflection, absorption) of the films was conducted using a spectral response measurement system (PerkinElmer Lambda 35 UV/VIS Spectrometer). The J-V curves were measured using a G2V optics small area Pico simulator at room temperature under AM 1.5 G illuminations (100 mW/cm^2^), and calibrated using a standard silicon solar cell device. An aperture mask of 0.09 cm^2^ was used to define the device area.

## 3. Results and Discussion

In our previous studies, we reported the effect of scCO_2_ annealing on the crystallinity and photovoltaic performance of perovskite devices [30,31,32]. The crystal structure was monitored using XRD and the diffraction peaks at 14.4°, 24.8°, 28.7°, and 32.2° correspond to the (110), (202), (220), and (310) planes of crystalline CH_3_NH_3_PbI_3_, respectively, with a tetragonal crystal structure [60,61,62,63]. The XRD pattern for PbI_2_ shows that, in general, thin film growth is oriented along the (001) direction at 12.73° irrespective of the spin parameters. This is typically due to the use of DMF, a less soluble, polar solvent containing electronegative oxygen atoms [64]. The presence of PbI_2_ typically indicates incomplete crystallization and/or degradation [65]. The effect of relative humidity on the CH_3_NH_3_PbI_3_ crystallinity was studied using XRD and UV-VIS.

**Figure 1:**
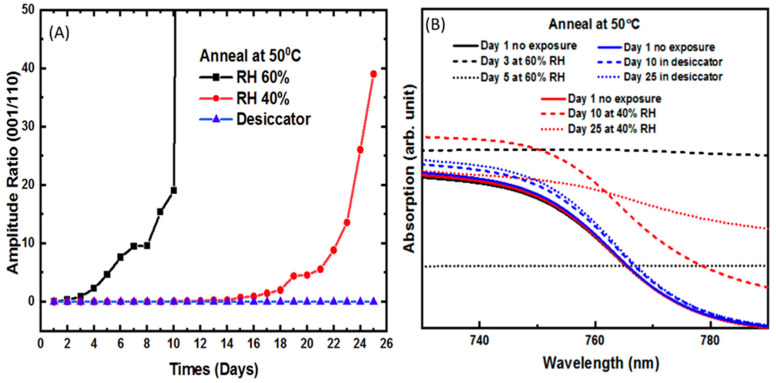
(**A**)Amplitude ratio of relative intensity of the (001) and (110) planes of CH_3_NH_3_PbI_3_ of films thermally annealed at 50 °C without scCO_2_. (**B**) Optical absorption spectra of the film at different humidity parameters corresponding to (**A**).

Figure 1, part A, shows the normalized amplitude ratio of the dominant PbI_2_ peak at (001) versus that of CH_3_NH_3_PbI_3_ (110) over 25 days after thermal annealing alone at 50 °C. An increase in this ratio is used as a measure of film degradation. Negligible degradation was observed in samples kept in a low humidity environment due to less moisture interactions with the perovskite film. However, when the samples were exposed to a relative humidity of 40%, there is an exponential decay beginning on about day 15. Exposing the thin films to 60% RH also shows rapid exponential degradation but beginning on the first day of exposure. Therefore, we observe an extremely strong correlation between an increase in relative humidity with degradation of the perovskite film.

Figure 1, part B, shows the optical absorption spectra of the perovskite film annealed at 50 °C at three different days for each relative humidity level. The absorption data is presented over a range of wavelengths near the perovskite band edge to try to understand the impact of moisture exposure on band-to-band transitions. Moisture damage will result in a loss of crystallinity and corresponding band structure. We, therefore, focus our attention on the shape of the band edge rather than the absorption intensity since the latter includes other factors such as film thickness and surface roughness, which were not independently measured. The low humidity sample shows very little change over time. However, at 40% RH, the spectrum begins to change on day ten and shows very little band edge by day 25. At 60% RH, the band edge disappears immediately leading to a featureless absorption spectra and by day five, we see no absorption spectra response.

**Figure 2:**
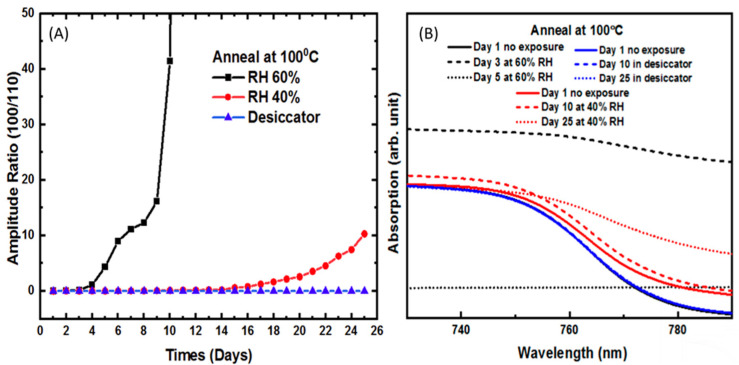
(**A**) Amplitude ratio of relative intensity of the (001) and (110) planes of CH_3_NH_3_PbI_3_ of films thermally annealed at 100 °C without scCO_2_ (**B**) Optical absorption spectra of the film at different humidity parameters corresponding to (**A**).

Figure 2, part A, shows the amplitude ratio of the PbI_2_ (001) peak to that of the CH_3_NH_3_PbI_3_ (110) peak versus time and at three different humidity levels for a sample annealed at 100 °C without scCO_2_. No degradation was observed for samples kept at low humidity. At 40%RH, no crystal degradation was observed until day 15, where the peak begins to increase slowly. At 60% RH, rapid, exponential degradation was observed beginning on day three. The slower degradation rate of films annealed at 100 °C compared to those annealed at 50 °C is likely due to the higher initial film quality and crystallinity in the films annealed at the higher temperature, as we reported previously.

Figure 2, part B, shows the optical absorption spectra of the perovskite film annealed at 100 °C without scCO_2_ at three different relative humidity levels. The absorption spectra for the dry sample remained unchanged after 25 days. At 40% RH, the absorption band edge is still clearly visible on day ten, but is significantly degraded by day 25. At 60% RH, the absorption band edge is almost entirely gone by day three and disappears entirely by day 5. This data confirms that traditional annealing at either 50 or 100 °C does not offer any significant protection against humidity degradation even though the films annealed at the higher temperature perform slightly better due to the superior initial crystallinity.

**Figure 3:**
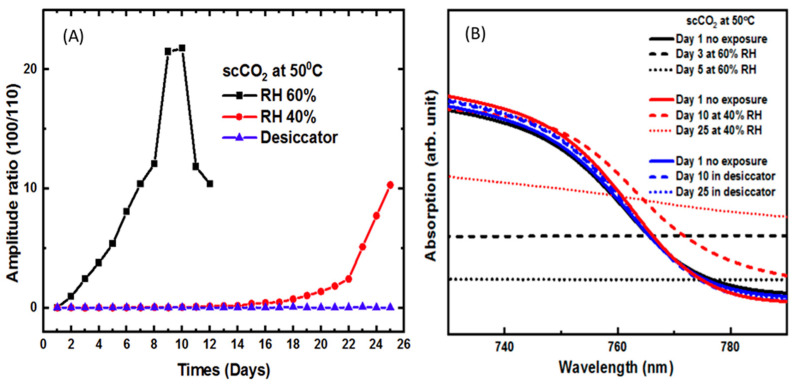
(**A**) Amplitude ratio of relative intensity of the (001) and (110) planes of CH_3_NH_3_PbI_3_ of films annealing at 50 °C in scCO_2_. (**B**) Optical absorption spectra of the film at different humidity parameters corresponding to (**A**).

Figure 3, part A, is a plot of the relative peak amplitude versus time for the sample processed in scCO_2_ at 50 °C at three different humidity levels. Negligible degradation was observed at the low humidity level. At 40% RH, degradation begins on the 15th day and increases slowly. At 60% RH, rapid exponential degradation was observed beginning on the first day of exposure, indicating that film degradation began immediately at this higher humidity level. After day nine, the absorption ratio plateaus and then decreases. We attribute this behavior to the very high level of film degradation. That is, after day nine, the crystal structure has been severely compromised and the X-ray diffraction data has little value.

Figure 3, part B, shows the optical absorption spectra of perovskite films processed in scCO_2_ at 50 °C and at three different humidity levels. The absorption spectra for the dry samples remains unchanged through the 25th day. At 40% RH, the band edge begins to change by day ten and is not discernable by day 25. At 60% RH, the band edge disappears completely by day three with no visible structure in the absorption spectra.

**Figure 4:**
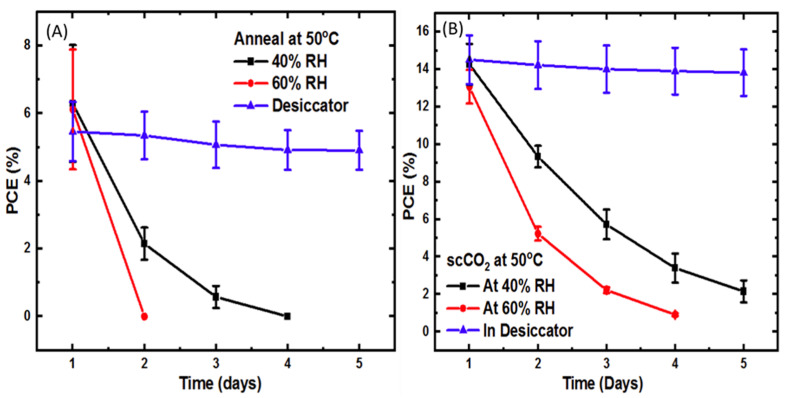
PCE versus time for perovskite films (**A**) annealed at 50 °C without scCO_2_ and (**B**) annealed at 50 °C in scCO_2_.

**Figure 5:**
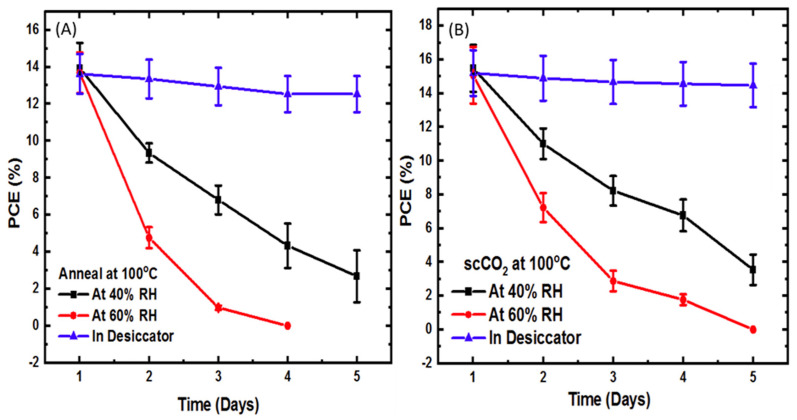
PCE degradation rate of perovskite films after annealing (**A**) at 100 °C without scCO_2_ and (**B**) at 100 °C with scCO_2_.

Figure 4 compares the power conversion efficiency versus time for films thermally annealed at 50 °C with (B) and without (A) scCO_2_ at three different relative humidity levels. Multiple photovoltaic devices were produced from each sample using a pixelated shadow mask to deposit the silver contacts. PCE data was obtained from each device and the error bars presented in Figure 4 and Figure 5 represent the precision intervals of the data. On day one (no humidity exposure), the average PCE of devices where the perovskite layer was annealed with scCO_2_ was about twice that of devices where the perovskite layer was annealed without scCO_2_. Specifically, devices where the perovskite layer was annealed at 50 °C had a PCE of 6.29 ± 1.72%, 6.12 ± 1.77%, and 5.46 ± 0.87%. However, devices where the perovskite layer was annealed at the same temperature, but in scCO_2_, had a PCE of 14.27 ± 1.09%, 13.07 ± 0.90%, and 14.52 ± 1.32%, respectively. This shows that perovskite films annealed in the presence of scCO_2_ exhibit much better photovoltaic performance at these lower temperatures due to superior film quality.

When the devices are kept in a dry desiccator, the PCE remains relatively constant, as would be expected. Upon exposure to RH levels of 40 and 60%, both sets of devices show a decrease in PCE with time, but the devices annealed in scCO_2_ exhibit a slower rate of degradation. Thus, perovskite films annealed in the presence of scCO_2_ at low temperatures not only exhibit superior film quality (greater smoothness, larger grain) and higher crystallinity, but also higher photovoltaic efficiency and a slower degradation rate upon exposure to humidity compared to films thermally annealed in the absence of scCO_2_ [30,31,32].

Figure 5 compares the power conversion efficiency versus time of devices, where the perovskite layer was thermally annealed at 100 °C with and without scCO_2_ and at three different relative humidity levels. At this higher temperature, the initial PCE and degradation rate at each RH level was approximately the same for devices annealed in scCO_2_ and devices annealed without scCO_2_. Therefore, we see no significant benefit in using scCO_2_ for annealing perovskite films at higher temperatures.

Comparing the photoconversion efficiency data of Figure 4 and Figure 5 to the X-ray and optical absorption data of Figure 1, Figure 2 and Figure 3 shows similar trends, but much different sensitivity. That is, the drop in observed device efficiency is consistent with the degradation in crystal structure and band structure, but is a much more sensitive indicator. For example, if we consider the films processed at 50 °C in supercritical CO_2_, the data of Figure 3, part A, at 40% RH shows the amplitude ratio increasing on day 14. However, the PCE data of Figure 4, part B, for devices constructed from these same films shows a sudden drop in PCE from 14% on day one to 9% on day two and down to 2% by day five. We conclude from this that device efficiency is a much more sensitive indicator of moisture degradation than either crystallographic or optical absorption measurements.

## 4. Conclusions

The influence of humidity on the rate of ${\mathrm{CH}}_{3}{\mathrm{NH}}_{3}$Pb$I_{3}$ perovskite film degradation was measured for films processed in supercritical CO_2_ at 50 and 100 °C in comparison to films annealed at the same temperatures but without supercritical CO_2_. The film quality over 25 days was measured for three different relative humidity levels using X-ray diffraction, optical absorption, and device photoconversion efficiency. For devices processed at 50 °C, it was determined that supercritical CO_2_ increases both the initial film quality as well as the resistance to humidity degradation and results in devices with higher photoconversion efficiency and a lower rate of performance degradation. For devices processed at 100 °C, no significant improvement in film quality, photoconversion efficiency or humidity resistance was observed for films processed in supercritical CO_2_.

## Data Availability

The data presented in this study are available on request from the corresponding author.

## Sample Availability

Samples of the compounds “perovskite films and devices” are available from the authors.
